# Letter to the editor: COVID-19: how accurate are seroprevalence studies?

**DOI:** 10.2807/1560-7917.ES.2020.25.30.2001374

**Published:** 2020-07-30

**Authors:** Kamran Kadkhoda

**Affiliations:** 1 Immunopathology Laboratory, Robert J. Tomsich Pathology & Laboratory Medicine Institute, Cleveland Clinic, Cleveland, OH, United States

**Keywords:** COVID-19, SARS-CoV-2, Prevalence, Serology, Antibody

**To the editor**: I read with great interest the study conducted by Percivalle et al. on seroprevalence of severe acute respiratory syndrome coronavirus 2 (SARS-CoV-2) in Lodi Red Zone, Lombardy, Italy [1]. While serosurveys are valuable public health tools in understanding, dissecting, and responding to outbreaks, they may still suffer from two main issues: (i) inadvertent biased design and/or (ii) suboptimal serological assays used.

In this study, 390 plasma specimens from apparently healthy (asymptomatic) blood donors collected from 18 March to 6 April 2020 were screened. The first two coronavirus disease (COVID-19) cases in Italy were detected in two Chinese tourists in Rome, on 31 January 2020 [2]. Subsequently, a cluster of cases was found in Lombardy on 21 February 2020 [3]. The authors in this study [1] reported a seroprevalence of 23% in Lodi, Italy among healthy blood donors, which given the current population, means ca 11,845 cases were in that small zone only 26 days after the first cluster was detected in Lombardy.

The authors of the study also showed that only 5% of these donors were positive for SARS-CoV-2 by RNA assay and only 3% of seropositive cases were also RNA positive. Given the delayed seroconversion associated with this virus, this would mean that the vast majority of the donors who tested positive by the neutralisation assay became infected at the very beginning of the outbreak in Lombardy.

On 8 July 2020, several months after this study is done, the total number of confirmed cases in Lombardy (population ca 10 million), Italy, was 94,651 [4]. Furthermore, according to the China–World Health Organization (WHO) joint report that was recently summarised in JAMA [5], 1–2% of cases are completely asymptomatic, and of symptomatic cases, ca 80% are mild or moderate. If we assume only the remaining 20% of the cases, i.e. severe and critical ones, are tested using RNA assays, the current seroprevalence is estimated to be around 4.7% in Lombardy. Since RNA testing has been expanded to more groups, the actual seroprevalence must even be lower than 4.7%. Had the authors used the samples from the very same donors in January 2020 and compared the test results with those of blood donations in March or April, by means of ≥ 4-fold rise in antibody titres, this would have shed more light on the true seroprevalence instead of using two different blood donor cohorts from two different time intervals.

It remains a possibility that recent exposure to common coronaviruses among donors caused a boost in the SARS-CoV-2 neutralisation assay used in this study. Neutralisation assay, although being a gold standard assay in the world of serological diagnosis, has its own limitations, cross-reactivity among others; this has been repeatedly shown in other settings, a recent example being dengue vs Zika virus neutralisation assays [6]. Cross-reactivity with common coronaviruses using neutralisation assays has also been shown [7]. Authors used 30 pre-pandemic samples to assess the specificity of their neutralisation assay without clarifying the time between symptom onset and sample collection for the common coronaviruses as antibody response to these viruses is short-lived. Had the blood donors in this study had a recent common coronavirus infection, this would have possibly affected their SARS-CoV-2 neutralisation assay results. Neutralisation assays are typically more specific when 90% inhibition of cytopathic effect with higher end titres is used as the cut-off. Using the cut-off titre of 10 with 50% inhibition of cytopathic effect, increases the sensitivity at the cost of lowering the specificity. This notion was further suggested in this study by showing nearly two third of the donors having had low neutralisation titres.

All in all, while it is appreciable that neutralisation assays are far more superior to other serological assays, and that cases are not evenly distributed in any given urban area, it is also pivotal to avoid under- and over-estimating the seroprevalence as it may have downstream consequences for public health measures, estimating the case fatality ratio and herd immunity, among others.
